# The Effects of L-Arginine on Liver Damage in Experimental Acute Cholestasis an Immunohistochemical Study

**DOI:** 10.1155/2011/306069

**Published:** 2011-06-01

**Authors:** Yucel Ozsoy, Mustafa Ozsoy, Teoman Coskun, Kemal Namlı, Ahmet Var, Beyhan Özyurt

**Affiliations:** ^1^Department of General Surgery, Manisa State Hospital, Manisa, 35630 İzmir, Turkey; ^2^Department of General Surgery, School of Medicine, Celal Bayar University, 45000 Manisa, Turkey; ^3^Department of Immunopathology, School of Medicine, Celal Bayar University, 45000 Manisa, Turkey; ^4^Department of Biochemistry, School of Medicine, Celal Bayar University, 45000 Manisa, Turkey; ^5^Department of Public Health and Biostatistics, School of Medicine, Celal Bayar University, 45000 Manisa, Turkey

## Abstract

Obstructive jaundice damages critical functions in the liver. Nitric oxide modulation would influence liver damage induced by biliary obstruction, and little is known about it Acute cholestasis was induced by bile duct ligation (BDL) in two groups of male Sprague-Dawley rats. L-Arginine or serum physiologic was administered to treatment and control group. Histopathological and immunohistochemical iNOS expression was investigated in hepatic tissue. Plasma enzyme activities were increased in acute cholestasis, and that L-arginine treatment partially but significantly prevented the elevation of these markers of liver damage (*P* <  .05). Also histopathology scoring showed that the liver injury was prevented and immunohistochemical iNOS activity was increased significantly in L-arginine group (*P* <  .05). This study shows that, after 7 days of biliary obstruction, liver damage is well established and exogenous L-arginine treatment partially but significantly prevented the liver injury in acute cholestasis.

## 1. Introduction

Biliary obstruction is a common surgical problem. Despite the advances in diagnostic and therapeutic modalities, its morbidity and mortality are high. Cardiovascular dysfunction, peripheral vasoconstriction, renal insufficiency, gastrointestinal hemorrhage, coagulopathy, and sepsis are major complications that affect its morbidity and mortality [1]. According to experimental and clinical studies, these complications develop because of hypotension, immunosuppression, hepatic dysfunction, and high serum levels of toxic substances such as bile salts and endotoxin [1]. Nitric oxide (NO), which appears as an intercellular and intracellular messenger, has a role in liver damage. The synthesis of NO from L-arginine is known to exist in a number of cells, including endothelial cells, macrophages, cerebellar neurons, neutrophils, Kupffer cells, and hepatocytes [2]. Macrophages and hepatocytes produce much greater quantities of NO, which is not detectable until several hours after exposure to a specific inflammatory or septic stimulus [3]. Although the roles of NO in liver damage induced by ischemia reperfusion [4], alcohol [5], dimethylnitrosamine [6], and immunological [7] liver injury have been studied, the function of NO in liver damage induced by bile duct ligation in the rat has not been ascertained.

This study was undertaken to determine if an alteration in NO production caused by external L-arginine administration would influence the degree of hepatic damage induced by a seven-day biliary obstruction.

## 2. Material and Methods

### 2.1. Animals

Thirty male Sprague-Dawley rats weighting 200–250 g were housed under standard laboratory conditions and allowed free access to food and water. Institutional guidelines for animal care were followed throughout the experimental study. The rats were anesthetized by intramuscular 50 mg/kg ketamine hydrochloride (Ketalar, Eczacıbaşı, Turkey) and 5 mg/kg xylazine hydrochloride (Rompun %2, Bayer). Extra hepatic cholestasis was induced by double ligation and section of the common bile duct with a 4/0 silk suture. Three groups of rats (*n* = 10) were used. The animals of the first group were sham operated. In the second group, animals were bile duct ligatured (BDL) and received 1 mL % 0.9 NaCl twice a day intraperitoneally (i.p.). Animals in the third group were BDL and given i.p. administration of 1 mL 500 mg/kg L-arginine twice a day. All the animals were sacrificed on the seventh day under ether anesthesia. Blood was collected by cardiac puncture, and the liver was rapidly removed. Small liver sections from the same lobe of liver from each rat were fixed in 10% neutral formalin, which was used for hematoxylin-eosin staining for histological and immunohistochemical examination under light microscopy.

### 2.2. Serum Enzyme Activities and Bilirubin Determination

Serum was also obtained for determination of aspartate aminotransferase (AST), alanine aminotransferase (ALT) [8], alkaline phosphatase (ALP) [9], and *γ*-glutamyl transpeptidase (*γ*GTP) [10, 11] activities. All of these markers, especially ALT, ALP, and *γ*GTP, are indicators of liver damage. Bilirubin content (kit from Mega, Merk, Germany) was studied for signs of cholestasis.

### 2.3. Histopathology and Immunocytochemistry

Liver histopathology was evaluated to show the effect of NO on cholestatic liver injury. Immunohistochemical study was performed to explain the effect of exogenous L-arginine on hepatic inducible nitric oxide syntheses (iNOS) expression. Histopathological studies were performed on 5-*μ*m slides. Inducible nitric oxide synthase (iNOS) was determined immunohistochemically by a standardized streptavidin-biotin-peroxidase method on formaline-fixed paraffin-embedded liver tissue. A quantitative method was used for immunohistochemical evaluation; the staining score was obtained by calculating the number of iNOS-stained epithelial cells per 100 asinus under 40x light microscopy. 

Histopathological evaluation was performed with a quantitative method. Ten different squares were used in each section to score the injury in the liver tissue. We used a modified scoring system for focal hepatic necrosis, as described by Orrego et al. [1], which takes into account necrosis, bile duct proliferation, and periportal leukocyte infiltration [12] (Tables 2 and 3), under 40x light microscopy (Olympus Bx40, Japan).

## 3. Results

All bilirubins increased progressively after bile duct obstruction and reached a plateau between three and seven days after surgery. Because of the cholestatic liver damage, plasma bilirubin levels and the activities of aspartate aminotransferase [8], alanine aminotransferase, alkaline phosphatase [9] and *γ*-glutamyl transpeptidase [10, 11] were all significantly higher in BDL rats than in sham-operated rats (*P* < .001, Table 1). 

L-arginine administration partially but significantly prevented the elevation of plasma enzyme activities in bile duct-ligated rats (*P* < .05, Table 1).

**Figure 1:**
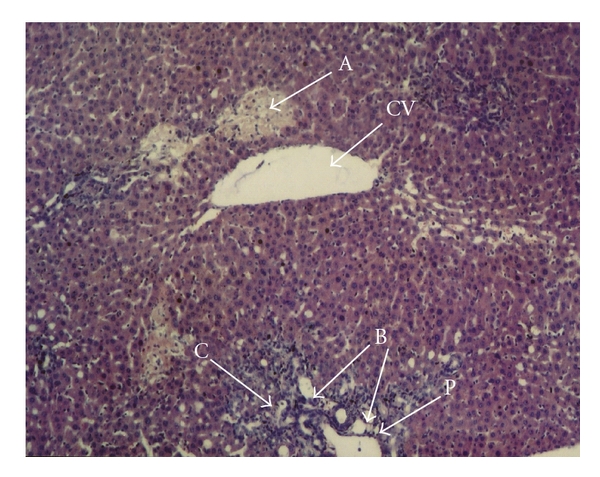
The liver histopathological changes in obstructive jaundice group, H-E ×40 Focal necrose areas (a), bile duct proliferation (b) and periportal PMN leucocytes infiltration (c). CV: central vein. P: portal vein.

**Figure 2:**
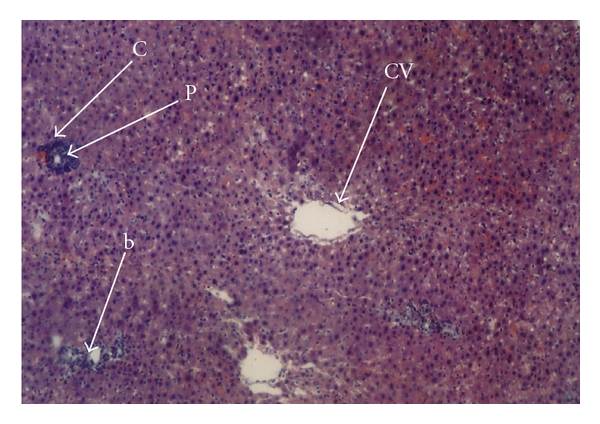
The liver histopathological changes in L-arginine group, H-E ×40. Less bile duct proliferation (b) and periportal PMN leukocyte infiltration (c) with no necrosis. CV: central vein. P: Portal vein.

 Histopathological studies showed significant differences between obstructive jaundice and L-arginine groups in hematoxylin-eosin-(H-E)-stained sections. In the obstructive jaundice group, swelling and vacuolization in the cytoplasm of hepatocytes, loss of eosinophilic structures, and nucleolus proliferation were seen. Dilatation of sinusoids due to periportal, perisinusoidal, and polimorpho- and mononuclear leukocyte infiltration was noticeable. Focal hepatic necrosis and periportal new bile canaliculi proliferation were also observed (Figure 1). These findings were less evident in the L-arginine treatment group in which there was minimal periportal, perisinusoidal, polimorpho- and mononuclear leukocyte infiltration. There were also less piknotic nucleus cells and necrosis in the exogen L-arginine-administered group (Figure 2) and (Tables 2 and 3).

**Figure 3:**
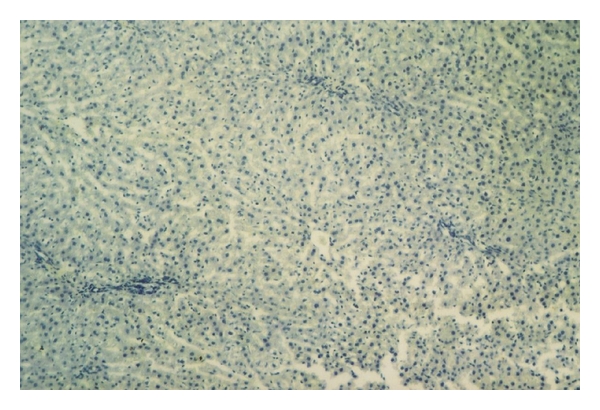
Sham group was not dyed by iNOS. (iNOS ×40).

**Figure 4:**
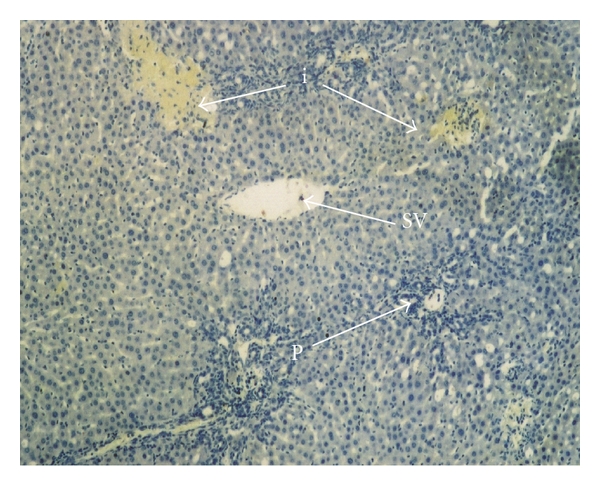
The liver iNOS staining in obstructive jaundice group, H-E ×40. A few areas iNOS staining (i), bile duct proliferation (b), and periportal PMN leucocytes infiltration (c). CV: central vein, P: portal vein (in the bile duct-ligated group, iNOS staining was observed most evidently in necrotic areas and also less in liver parenchyma).

Immunohistochemical staining was evaluated in the hepatocytes using cytoplasmic patterns. In the sham-operated group, iNOS staining was almost absent in liver parenchyma and periportal areas (Figure 3). In the bile duct-ligated group, iNOS staining was most evident in necrotic areas, and observed to a lesser extent in liver parenchyma (Figure 4).

**Figure 5:**
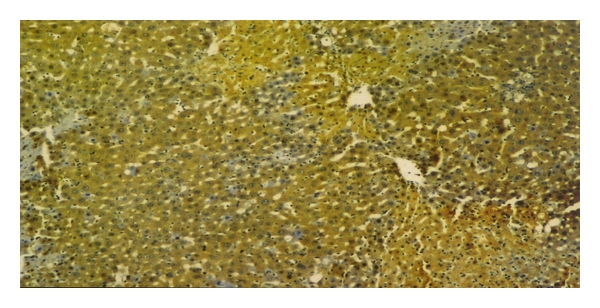
The liver iNOS staining increased in L-arginine group, H-E ×40. Diffuse iNOS staining in L-arginine group.

 In the same areas in the obstructive jaundice + L-arginine group, iNOS staining intensity increased with L-arginine administration, and focal necrosis, bile duct proliferation, and periportal leukocyte infiltration were partially, but significantly, (*P* < .001) prevented (Figure 5). The iNOS tissue staining scores were 15.85% in the sham-operated group, 32.70% in the BDL group, and 65.22% in the obstructive jaundice + L-arginine group; there were significant differences between the obstructive jaundice and L-arginine groups.

## 4. Discussion

In this work, we have studied the effects of exogenous L-arginine on the production of NO by regulating the expression of iNOS proteins. We have also studied the effect of exogenous arginine on cholestatic liver injury. Recent observations provide evidence that NO plays an important role in liver disease, but little is known about the role of arginine-iNOS pathways in cholestatic liver injury [3, 13]. Bile duct ligation in the rat is a good model to study drugs that might prevent or reverse liver injury [3]. In this study's experiments, we have shown that, after seven days of biliary obstruction, liver damage was well established and is accompanied by decreased production of iNOS protein and elevated liver enzymes. As a consequence of the decrease in iNOS synthesis, liver blood supply and perfusion decreased. Ischemia and necrosis were observed in the liver under light microscopy [14]. 

Recent researches about the role of NO in the regulation of hepatic microcirculation have shown that the L-arginine-derived iNOS activation supports vasodilatation and inhibition of platelet functions. NO may exhibit its protective effects against liver injury through vasodilatation and inhibition of platelet and macrophage activities [15, 16]. In addition, studies in cholestatic rats have shown an association with hepatic ischemia because of the deficient production of iNOS and protein. This supports the hypothesis that NO regulates blood flow in cholestatic rats [17, 18]. 

However, different results were shown for the production of NO in acute cholestasis. While several studies have suggested that there is overproduction of NO in their experimental models of bile duct ligation [19], the results of other studies about cholestasis do not support this hypothesis [13] because they do observe the inhibition of NO production in cholestasis. Also, endogen iNOS activity was not increased, even in portal-hypertensive and cirrhotic rats [20, 21]. The same is true for BDL rats, as we have shown immunohistochemically in the current study [13].

In the current study, after exogenous L-arginine administration to cholestatic rats, iNOS expression and nitric oxide production were significantly increased and shown immunohistochemically for the first time. There is no previous study that has investigated the effects of exogenous L-arginine on liver iNOS expression and histopathological changes in acute cholestasis. Exogenous L-arginine is important because several studies have shown that L-arginine utilization is increased during obstructive cholestasis or endotoxemia [22, 23], and exogenous L-arginine administration partially but significantly prevented the elevation of liver enzymes. However, there are no studies about the histopathological findings of exogenous L-arginine on cholestatic liver injury. Our study showed that histopathological changes, such as focal necrosis, new bile canaliculi proliferation, and periportal leukocyte infiltration, were partially but significantly prevented by exogenous L-arginine in obstructive jaundiced rats. L-arginine played a protective role by inducing iNOS expression and NO synthesis, and this is also shown histopathologically and immunohistochemically for the first time. 

Muriel and González [3] and [23] have shown the effects of exogenous L-arginine in BDL rats with liver enzymes with markers of liver damage. Muriel suggested that L-arginine-induced NO production plays a protective role in cholestatic liver injury induced by 3-day biliary obstruction and showed this through decreased liver enzyme levels and lipid peroxidation, but further studies are needed to show the protective effects of exogenous L-arginine with histopathological findings and specific markers of liver injury [23]. The current experimental study has demonstrated that exogenous L-arginine partially but significantly decreases the liver enzyme levels and histopathological liver injury. 

In summary, the present study suggests that L-arginine treatment partially but significantly prevents the liver injury in acute cholestasis by stimulation of iNOS expression and NO synthesis. This is shown histopathologically and immunohistochemically for the first time.

## Figures and Tables

**Table 1:** Comparison of aspartate aminotransferase, alanine aminotransferase, alkaline phosphatase, and *γ*-glutamyl transpeptidase levels in sham-operated, obstructive jaundice and L-arginine groups.

Group	T. bilirubin	D. bilirubin	AST	ALT	ALP	GGT
	U/L	U/L	U/L	U/L	U/L	U/L
Sham (saline)	0.27 ± 0.3	0.02 ± 0.01	131 ± 23	90 ± 36	224 ± 42	2 ± 2
Obstructive jaundice (saline)	10 ± 3.0	8.00 ± 2.00	763 ± 88^a^	233 ± 74^a^	533 ± 11^a^	25 ± 11^a^
L-Arginine	7 ± 2.0	6.00 ± 2.00	347 ± 22^b^	141 ± 71^b^	365 ± 9^b^	15 ± 4^b^

Data are shown as means ± S.E.M.; ten rats were used in each group.

^a^ *P* < .05 Mann-Whitney *U*-test in comparison with sham (saline) group.

^b^ *P* < .05 Mann-Whitney *U*-test in comparison with obstructive jaundice (saline) group.

**Table 2:** Cholestatic liver injury scale.

Necrosis	
No necrosis	(0 point)
Necrosis in one and/or two cells	(1 point)
Focal necrosis that involves more than two cells	(2 point)
Massive confluent necrosis	(3 point)
Zonal massive necrosis and bridging necrosis between central veins	(4 point)

Bile duct proliferation	

1-2 bile canaliculi proliferation	(1 point)
3-4 bile canaliculi proliferation	(2 point)
>4 bile canaliculi proliferation	(3 point)

Periportal leukocyte infiltration	

Mild infiltration with one or two cells	(1 point)
Moderate infiltration with more than two cells	(2 point)
Diffuse infiltration	(3 point)

**Table 3:** Cholestatic liver injury score.

	Obstructive jaundice group	L-arginine group
Focal necrotic areas	2.11	0.22^a^
Ductus proliferation	2.7	1.44^a^
Periportal leukocyte infiltration	2.4	1.22^a^

Total score	7.2	2.8^a^

^a^ *P* < .001, Mann-Whitney *U*-test, in comparison with cholestatic (saline) group.
