# Erratum to "The Effects of L-Arginine on Liver Damage in Experimental Acute Cholestasis an Immunohistochemical Study"

**DOI:** 10.1155/2013/492846

**Published:** 2013-02-04

**Authors:** Yucel Ozsoy, Teoman Coskun, Yavuz Kaya, Kemal Ozbilgin, Ahmet Var, Beyhan Ozyurt

**Affiliations:** ^1^Department of General Surgery, Manisa State Hospital, 35630 Manisa, Izmir, Turkey; ^2^Department of General Surgery, School of Medicine, Celal Bayar University, 45000 Manisa, Turkey; ^3^Department of Histology and Embryology, School of Medicine, Celal Bayar University, 45000 Manisa, Turkey; ^4^Department of Biochemistry, School of Medicine, Celal Bayar University, 45000 Manisa, Turkey; ^5^Department of Public Health and Biostatistics, School of Medicine, Celal Bayar University, 45000 Manisa, Turkey

The authors' list is regarded as shown above. 

